# Disruption of myofibroblastic Notch signaling attenuates liver fibrosis by modulating fibrosis progression and regression

**DOI:** 10.7150/ijbs.60056

**Published:** 2021-05-27

**Authors:** Zhensheng Yue, Zijian Jiang, Bai Ruan, Juanli Duan, Ping Song, Jingjing Liu, Hua Han, Lin Wang

**Affiliations:** 1 Department of Hepatobiliary Surgery, Xi-Jing Hospital, Fourth Military Medical University, Xi'an 710032, China.; 2 Department of Ophthalmology, Xi-Jing Hospital, Fourth Military Medical University, Xi'an 710032, China.; 3 Aerospace Clinical Medical Center, School of Aerospace Medicine, Fourth Military Medical University, Xi'an 710032, China.; 4 State Key Laboratory of Cancer Biology, Fourth Military Medical University, Xi'an 710032, China.; 5 Department of Biochemistry and Molecular Biology, Fourth Military Medical University, Xi'an 710032, China.

**Keywords:** Myofibroblasts, hepatic stellate cells, Notch signaling, liver fibrosis progression, liver fibrosis regression

## Abstract

The phenotypic transformation of hepatic myofibroblasts (MFs) is involved in the whole process of the progression and regression of liver fibrosis. Notch signaling has been demonstrated to modulate the fibrosis. In this study, we found that Notch signaling in MFs was overactivated and suppressed with the progression and regression of hepatic fibrosis respectively, by detecting Notch signaling readouts in MFs. Moreover, we inactivated Notch signaling specifically in MFs with Sm22α^CreER^-RBPj^flox/flox^ mice (RBPj^MF-KO^), and identified that MFs-specific down-regulation of Notch signaling significantly alleviated CCl_4_-induced liver fibrosis during the progression and regression. During the progression of liver fibrosis, MFs-specific blockade of Notch signaling inhibited the activation of HSCs to MFs and increases the expression of MMPs to reduce the deposition of ECM. During the regression of fibrosis, blocking Notch signaling in MFs increased the expression of HGF to promote proliferation in hepatocytes and up-regulated the expression of pro-apoptotic factors, Ngfr and Septin4, to induce apoptosis of MFs, thereby accelerating the reversal of fibrosis. Collectively, the MFs-specific disruption of Notch signaling attenuates liver fibrosis by modulating fibrosis progression and regression, which suggests a promising therapeutic strategy for liver fibrosis.

## Introduction

Liver fibrosis is a highly conservative response to liver injury, which occurs in almost all types of liver injury. Liver fibrosis can be observed in chronic viral hepatitis, alcoholic liver disease, non-alcoholic fatty liver disease, cholestatic and autoimmune liver disease 1, 2. Under the continuous stimulation of harmful factors, the non-parenchymal cells, mainly hepatic stellate cells (HSCs), can be activated into myofibroblasts (MFs) and start expressing α-smooth muscle actin (α-SMA) and collagens to produce excessive extracellular matrix (ECM), thereby resulting in liver fibrosis 3, 4. However, when harmful stimuli are evacuated, the liver can undergo rapid regeneration, which is mainly manifested by the proliferation of hepatocytes under the action of mitogens, including Wnt2a, Wnt9b and hepatocyte growth factor (HGF) 5, 6. Moreover, a variety of cells including MFs and macrophages in the liver can produce matrix metalloproteinases (MMPs) that degrade ECM and fibrotic septum. During liver fibrosis regression, MFs convert into different cell fates, including inactivation, senescence and apoptosis 7-9, on the absence of pro-fibrotic factors, such as transforming growth factor-β (TGF-β), platelet-derived growth factor β (PDGF-β) and interleukin-17A (IL-17A), etc. 10-12. Additionally, the activation of death receptor-mediated pathways, the increase of pro-apoptotic proteins and the decrease of pro-survival proteins are considered to play causative roles in MFs apoptosis 7. MFs also increase the expression of Fas receptors or tumor necrosis factor receptor (TNFR) family members, which triggers caspase8/caspase3-dependent cell apoptosis. Moreover, overexpression of pro-apoptotic proteins such as p53 or Bax in MFs lead to caspase-mediated programmed cell death 13-15. In addition, MFs are also the main source of tissue inhibitors of metalloproteinases (TIMPs). The loss of MF during liver fibrosis regression can lead to decreased TIMPs expression, which in turn contributes to increased activity of MMPs and subsequent degradation of ECM 6, 16.

Notch signaling is a highly evolutionarily conserved pathway that regulates cell differentiation, proliferation and apoptosis in many kinds of tissues 17, 18. Activation of Notch signaling relies on binding of Notch receptors (Notch1, -2, -3, and-4) and ligands (Jagged1, -2 and Dll1, -3, -4) between neighboring cells. This ligand receptor interaction triggers sequential receptor cleavage within the transmembrane domain, resulting in the release of the Notch intracellular domain (NICD). NICD then transfers into the nucleus and combines with transcription factor CSL (C promoter-binding factor 1[CBF-1]/suppressor of hairless [Su(H)]/Lin-12 and Glp-1 [LAG-1], also known as j-kappa recombination signal-binding protein [RBPj] in mice), which is inhibited by a corepressor complex under normal conditions. Association of NICD and CSL replaces the corepressors with a coactivating complex containing Mastermind-like (MAML) protein and activates the transcription of downstream genes, such as Hes and Hey family members 19, 20.

Notch signaling pathway participated in fibrosis in a variety of tissues and organs, such as skin, kidney, lung and liver 21-24. It was initially found *in vitro* that rat HSCs express Notch receptors and begin expressing Jagged1 upon activation and differentiation to MFs 25. Afterwards, other *in vivo* studies showed that Notch signaling pathway can also cooperate with other signaling pathways to mediate the activation process of HSCs to MFs, such as TGF-β, Wnt and Hedgehog signaling pathways 26, 27. Our previous studies found that myeloid-specific disruption of RBPj ameliorates hepatic fibrosis by inhibiting the activation of HSCs to MFs and liver inflammation and reducing the expression of pro-fibrotic factors, including TGF-β and PDGF-β 28. Moreover, endothelial-specific Notch signaling activation can aggravate liver fibrosis 29. The *in vivo* dependence of HSCs activation on Notch signaling indicates that inhibition of this pathway in the liver can prevent fibrosis or significantly ameliorate fibrosis 30. However, the overall consequence of MFs-specific Notch inactivation during fibrosis progression and regression has not been evaluated. In this study, we report that MFs-specific disruption of Notch signaling attenuates liver fibrosis by inhibiting fibrosis progression and altering MFs expression spectrum that expedited fibrosis regression.

## Materials and Methods

### Animal experiments

C57BL/6 wild-type mice were from the Laboratory Animal Center of Air Force Military Medical University, Sm22α^CreERT2^ mice (Model Animal Institute of Nanjing University) were crossed with RBPj^flox/flox^ mice (Jackson Labs). The littermates were genotyped by PCR to obtain Sm22α^CreERT2^ (control, Ctrl) and Sm22α^CreERT2^-RBPj^flox/flox^ mice. Male Sm22α^CreERT2^-RBPj^flox/flox^ mice of 6-8 weeks old were injected with carbon tetrachloride (CCl_4_, mixed with olive oil, 0.6 mL/kg, twice a week, Sigma-Aldrich) and tamoxifen (100 mg/kg, 5 times started from the fifth week, Sigma-Aldrich, St. Louis, MO) intraperitoneally (IP) to obtain RBPj^MF-KO^, with olive oil as a control. Progressive liver fibrosis mice model and regressive liver fibrosis mice model: liver fibrosis progression mice were induced by intraperitoneal CCl_4_ injection for 6weeks as previously described 31. For fibrosis regression study, the CCl_4_ injections were terminated after 6 weeks injection for 5 days as previously described 31.

All the animals were maintained in the specific pathogen free (SPF) level standards environment. All the operations of the experimental animals were reviewed the Animal Experiment Administration Committee of the Fourth Military Medical University (Xi'an, China) and were under China National “Regulations on the Management of Experimental Animals”. The study complied with the guideline outlined in the National Institutes of Health Guide for the Care and Use of Laboratory Animals of the National Academy of Science and published by the National Institute of Health.

### Histopathology

Mice liver samples were fixed in 4% paraformaldehyde (PFA) or in 10% buffered formalin. PFA-fixed samples were embedded with optimal cutting temperature (OCT) compound and sectioned at 8-μm thickness for immunofluorescence (IF) as described previously. Immunofluorescence (IF) was performed using fluorescent antibodies and counterstained with Hoechst 33258 (Sigma-Aldrich). Formalin-fixed samples were paraffin-embedded, sectioned, and subjected to hematoxylin and eosin (HE) staining and immunohistochemistry (IHC) routinely. Apoptotic cells were stained with a terminal deoxynucleotidyl transferase-mediated dUTP nick end labeling (TUNEL) kit (Promega, Madison, WI). Specific antibodies shown in Supplementary Table 2. Digital images were taken under a fluorescence microscope (BX51; Olympus, Japan) or laser-scanning confocal fluorescence microscope (FV-1000; Olympus, Japan). Transmission electron microscopy (TEM) and scanning electron microscopy (SEM) were carried out as described previously.

### Cell isolation, culture, and identification

Primary HSCs/MFs were isolated from mice by *in situ* perfusion followed by finely minced with a tissue homogenizer in perfusion buffer containing Dnase I (Roche, Basel, Switzerland)/Collagenase IV (Sigma-Aldrich) and subsequent density gradient centrifugation as described previously. The obtained cells were identified by α-SMA and Collagen1 IF staining and purity was more than 95%. The isolated primary HSCs/MFs were then cultured in low glucose Dulbecco's modified Eagle's medium (DMEM) (Gibco, USA), supplemented with 10% fetal bovine serum (FBS) (Biological Industries, Israel), and maintained in 37 °C incubator with 5% CO_2_. Culture medium was replaced every 24 h. Human HSCs cell line LX-2 (kindly provided by YM-Li, State Key Laboratory of Cancer Biology, FMMU, China) was cultured in high glucose DMEM (Gibco, USA), supplemented with 10% FBS and maintained in 37 °C incubator with 5% CO_2_. GSI (Sigma Aldrich) and TGF-β (Pepro Tech) were added at concentrations of 100 μM and 0.1 ng/mL respectively.

### Gene expression profiling

The total RNA of fresh isolated and purified MFs from RBPj^MF-KO^ and control mice was extracted by using TRIzol (Invitrogen), according to the manufacturer's protocol. Guangzhou GENE DENOVO Biology (Co. Ltd.) was commissioned to carry out quality inspection and full transcriptome expression profile sequencing service, using Digital Gene Expression Tag Profiling mode sequencing. Bioinformatics analysis such as KEGG signal pathway enrichment analysis, GO enrichment analysis, Heat Map and other bioinformatics analysis were carried out by using OmicShare Tools platform (www.omicshare.com/tools).

### qPCR

Total RNA extraction and reverse transcription were performed by using TRIzol and PrimeScrip RT reagent kit (TaKaRa Biotechnology, Dalian, China) according to standard procedures. qPCR was performed using a TB Green Premix Ex Taq Kit (TaKaRa Biotechnology) and an ABI PRISM 7500 Real-time PCR system (Life Technologies, Waltham, MA), with β-Actin as a reference control. The primers are showed in Supplementary Table 1.

### Western blot analysis

Total Protein was extracted from liver tissue or MFs with RIPA (Beyotime) lysis buffer containing phosphatase inhibitor (phenylmethylsulphonyl fluoride, PMSF), and protein concentration was determined by Pierce BCA Protein Assay Kit (Thermo Scientific, USA). Following separation by sodium dodecyl sulfate-polyacrylamide gel electrophoresis (SDS-PAGE), proteins were blotted onto polyvinylidene fluoride (PVDF) membranes, and probed with specific antibodies shown in Supplementary Table 2. Protein bands were detected using an enhanced chemiluminescence (ECL) system, with β-Actin or GAPDH as a loading control.

### Hydroxyproline test

Hydroxyproline content was quantified to determine the extent of liver fibrosis by using a kit (Sigma- Aldrich) according to the manufacturer's instruction. Briefly, collected flash frozen liver samples were weighed, homogenized and hydrolyzed in concentrated hydrochloric acid (HCl, 12N) in pressure-tight vials with PTFE-lined cap at 120°C for 3 hours. Equivalent volume of supernatant was transferred to a 96 well plate and was evaporated to dryness under a vacuum. Chloramine T/oxidation buffer mixture was added to each well and incubated at room temperature for 5 minutes. Next, diluted 4-(Dimethylamino) benzaldehyde reagent was added to each sample and was incubated at 60 °C for 90 minutes. Absorbance at the wavelength of 560 nm was determined, and the content of hydroxyproline in the liver was calculated on the basis of the sample weight by comparing the absorbance to a hydroxyproline standard curve.

### Statistics analysis

The Image-Pro Plus 6.0 program was used to carry out morphological quantitative. Statistical analysis was performed with the GraphPad Prism 6 software. All the results were presented as the mean ± standard deviation (SD). Comparisons between groups were performed using unpaired, two-tailed, Student's t-test. *P* < 0.05 was considered statistically significant.

## Results

### Notch signaling pathway is activated in liver fibrosis progression and suppressed in liver fibrosis regression

**Figure 1:**
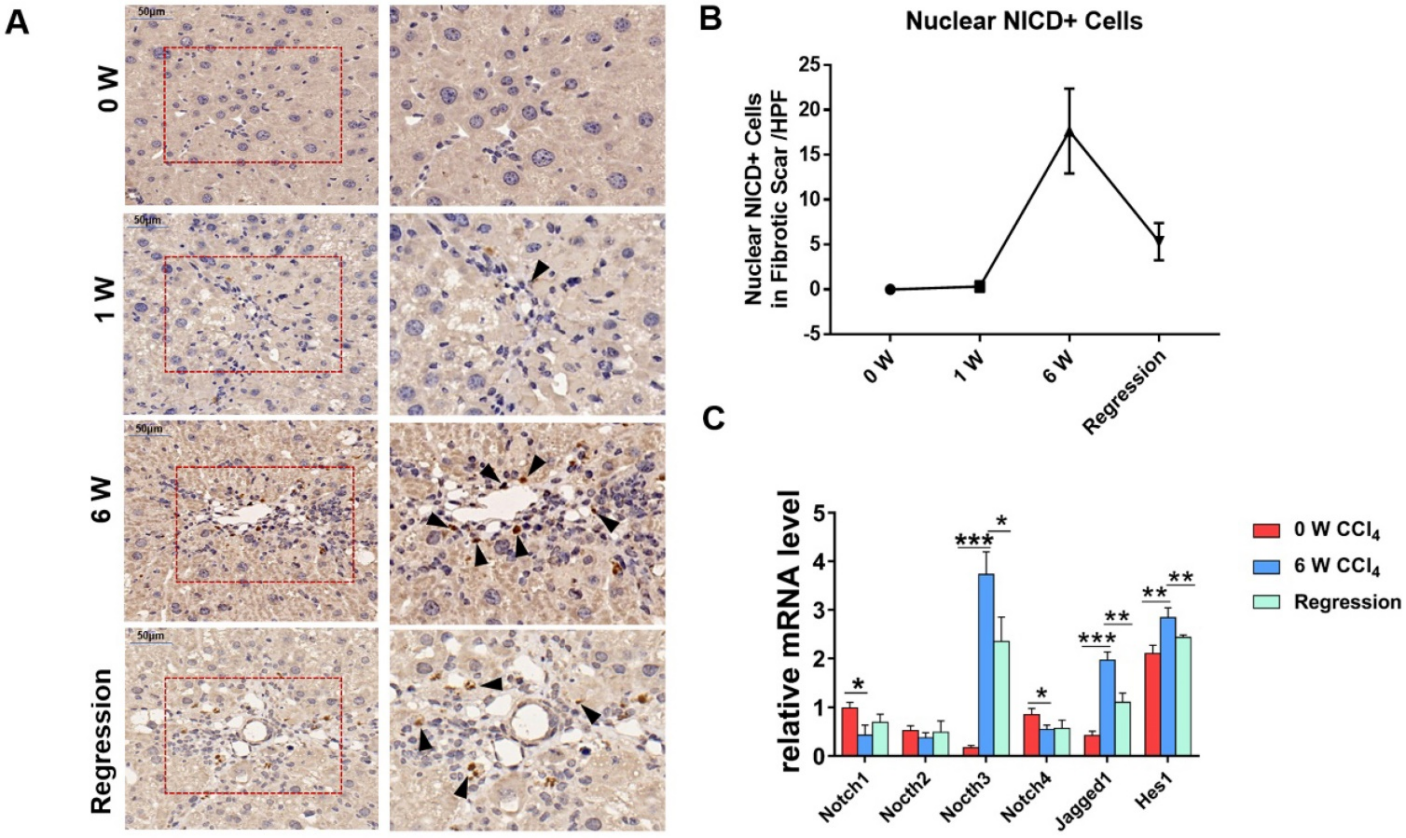
** The Notch signaling pathway in MFs participates in the regulation of liver fibrosis progression and regression.** The samples of olive oil or CCl_4_ injected C57BL/6 mice for 0, 1, 6 weeks and regression for 5 days were subjected to the following assays. (A) NICD immunohistochemical staining of liver fibrous scar area in different stages of liver fibrosis. (B) The number of nucleus NICD positive cells in the fibrous scar area increases with progression, and decreases with regression. (C) Changes in the expression of Notch signaling related molecules in liver HSCs/MFs at different stages of liver fibrosis. (Bars = means ± SD, n = 6, *P <0.05, **P < 0.01, ***P < 0.001).

To investigate whether Notch signaling mediates the liver fibrosis, we detected the expression of Notch signaling readouts in MFs. IHC staining showed the translocation of NICD into the MFs nucleus in fibrosis septum area (Figure 1A). Statistical analyses indicated that the number of nuclear NICD positive cells in the fibrous scar area increases with progression, and decreases with regression (Figure 1B). Then, we isolated the HSCs/MFs of wild-type mice in different stages of liver fibrosis. The results of qPCR showed that the expression of Notch3, Jagged1 increased with the progression of hepatic fibrosis and decreased with the reversal of hepatic fibrosis, consistent with previous reports 32-34. Although the expression of Notch1 and Notch4 decreased during the progression of hepatic fibrosis, the expression pattern of Hes1, a target gene downstream of Notch signaling, was similar to Notch3 and Jagged1, fluctuated with the progression and regression of hepatic fibrosis (Figure 1C). These results indicated that Notch signaling closely correlates with liver fibrosis and may be involved in the progression and regression of liver fibrosis.

### Induction of liver fibrosis mice and MFs-specific Notch disruption mice (RBPj^MF-KO^)

**Figure 2:**
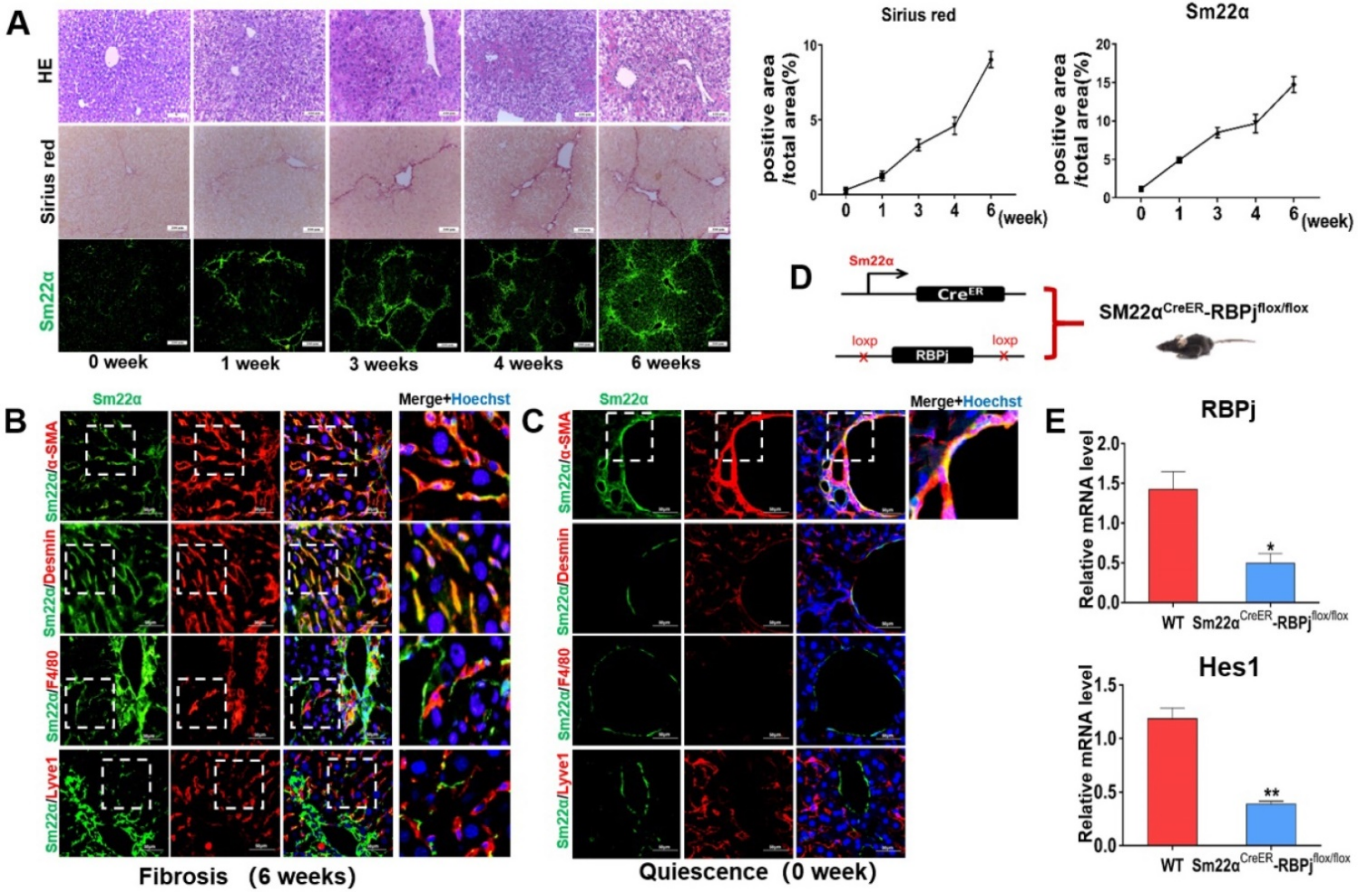
** Establishment and identification of RBPj^MF-KO^ models of liver fibrosis.** (A) Representative hematoxylin-eosin (HE)-stained liver sections from mouse exposed to CCl_4_ for 1, 3or 6 weeks for evaluation of steatosis. Sirius red staining for evaluation of collagen deposition. Sm22α immunofluorescence staining for evaluation of activation of HSCs. (B) In liver fibrosis, Sm22α is mainly expressed in MFs. (n=6). (C) In normal liver, Sm22α is only expressed in vascular smooth muscle cells (n=6). (D) Induction schedules of genetically modified mouse models. (E) qPCR showed the expression changes of Notch signaling downstream molecules, including RBPj and Hes1, in isolated MFs of Sm22α^CreER^- RBPj^flox/flox^ mice after CCl_4_ and tamoxifen induced. (Bars = means ± SD, n=3, *P < 0.05, **P < 0.01, ***P < 0.001).

Liver fibrosis was induced by repeated intraperitoneal injection of CCl_4_ for 6 weeks 29. HE and Sirius red staining of liver tissues showed that the area of centrilobular necrosis was increased accompanied by bridging fibrous septa in the livers of CCl_4_-treated mice (Figure 2A). In a systemic approach to obtain the MFs-specific Notch disruption mice, we detected the expression of transgelin (Smooth muscle 22α, Sm22α) in liver (Figure 2A). Previous studies have shown that Sm22α is not expressed in quiescent HSCs, but gradually up-regulated during the activation of HSCs 32-34. In our research, Sm22α IF staining showed that the expression of Sm22α increased gradually with the increase of CCl_4_ injection times, and was abundant at 3-4 weeks (Figure 2A). In order to precisely identify the expression of Sm22α in hepatic nonparenchymal cells, we compared the expression of Sm22α with α-SMA, Desmin, F4/80 and Lyve1, other kinds of nonparenchymal cells markers in the liver. The results of co-localization staining showed that in the fibrotic liver, Sm22α had a high proportion (96%) of co-localization with α-SMA and Desmin (Figure 2B). But in normal liver, Sm22α is only co-located with α-SMA around large vessels (Figure 2C). This indicates that during hepatic fibrosis, Sm22α is mainly expressed in MFs while Sm22α is only expressed in perivascular smooth muscle cells in normal liver. Based on the expression of Sm22α, we hybridized Sm22α^CreER^ mice and RBPj^flox/flox^ mice and screened out Sm22α^CreER^-RBPj^flox/flox^ from the descendants (Figure 2D). In CCl_4_- and tamoxifen-induced Sm22α^CreER^-RBPj^flox/flox^ mice, the expression of RBPj and Hes1, downstream molecules of canonical Notch signaling, was down-regulated significantly in MFs isolated from Sm22α^CreER^-RBPj^flox/flox^ mice (Figure 2E), indicating that we obtained MFs-specific Notch disruption mice (RBPj^MF-KO^).

### MFs-specific Notch signaling disruption inhibits the progression of hepatic fibrosis via decreasing ECM deposition and liver tissue destruction

**Figure 3:**
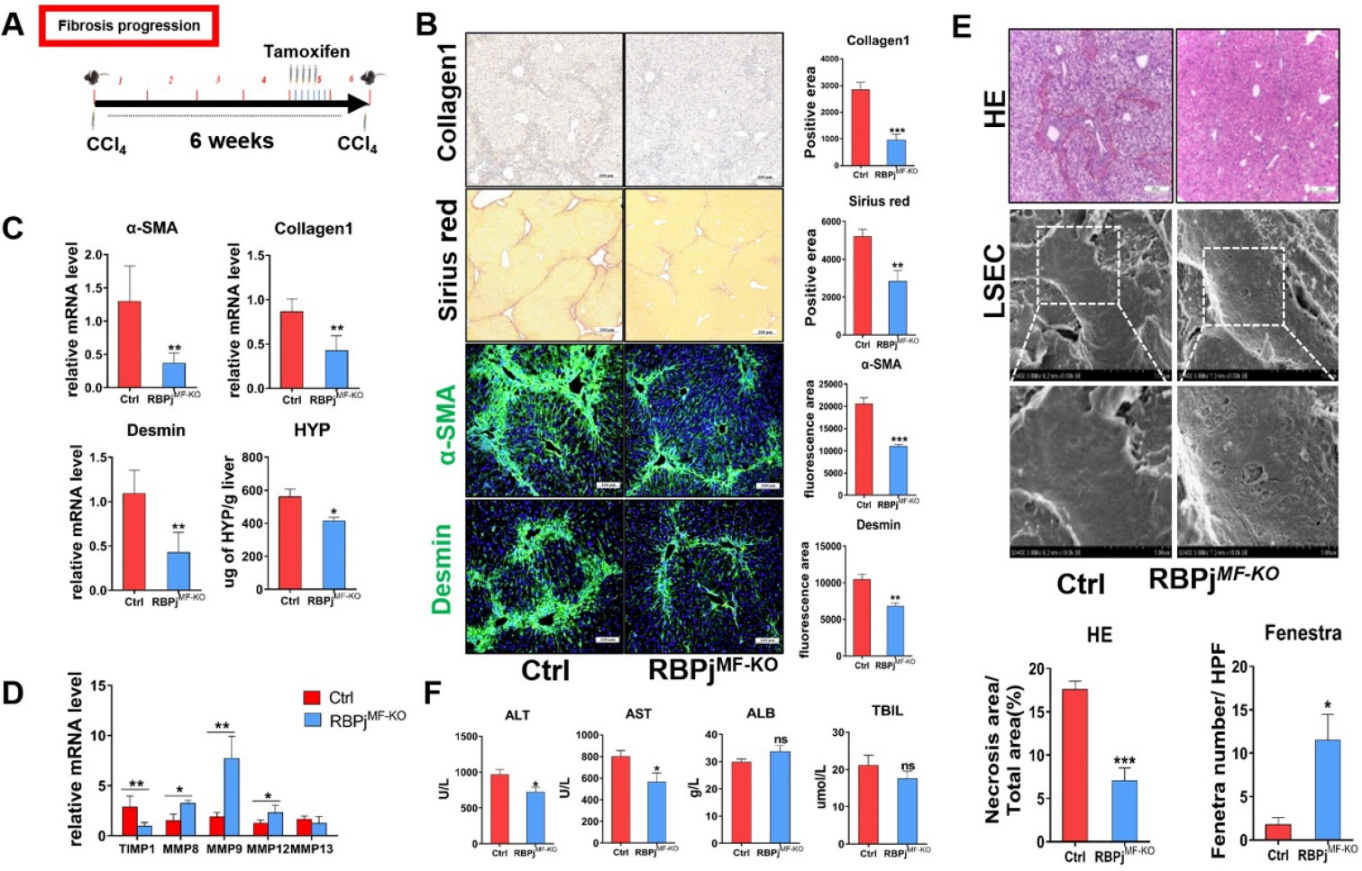
** Blocking of Notch signaling in MFs inhibits the progression of CCl_4_-induced liver fibrosis.** (A) Knockout induction schedule for liver fibrosis progression models. (B) Liver Collagen1, Sirius red, α-SMA, Desmin staining showed ECM was significantly reduced. (C) Changes in mRNA expression of liver tissue fibrosis-related molecules and changes in liver hydroxyproline level. (D) Changes in the expression of TIMP1 and MMPs in liver tissues. (E) HE staining of liver tissue and SEM of LSEC. The statistical results of HE staining showed that the destruction of liver tissue was reduced; the statistical results of fenestrae showed that the degree of capillarization of hepatic sinusoid was reduced. (F) The serum ALT and AST level of RBPj^MF-KO^ mice decreased. (Ctrl=5, KO=6, *P < 0.05, **P < 0.01, ***P < 0.001, ns, not significant).

In fibrosis progression mouse models, CCl_4_ and tamoxifen were injected as previously reported (Figure 3A). Induction of liver fibrosis progression in RBPj^MF-KO^ and control mice showed that RBPj^MF-KO^ mice manifested significantly alleviative fibrogenesis and decreased ECM deposition, as evidenced by less intensely stained Collagen1 and Sirius Red (Figure 3B) and reduced tissue hydroxyproline (HYP) level (Figure 3C). IF staining showed Notch inactivation in MFs down-regulated α-SMA and Desmin expression in liver (Figure 3B). qPCR showed that the mRNA level of Collagen1, α-SMA and Desmin also decreased in RBPj^MF-KO^ liver (Figure 3C). Additionally, mRNA level of MMP8/9/12 elevated and TIMP1 reduced in RBPj^MF-KO^ liver (Figure 3D). To confirm the condition of liver tissue destruction, liver sections from RBPj^MF-KO^ and control mice were analyzed with HE staining and SEM. In RBPj^MF-KO^ mice of fibrosis progression, less necrosis area and more liver sinusoid fenestrae were observed, suggesting alleviated liver tissues destruction and liver sinusoid capillarization (Figure 3E). Additionally, serum ALT and AST level of RBPj^MF-KO^ mice was lower than control mice though ALB and TBIL level didn't change significantly (Figure 3F). These data collectively indicated that blocking Notch signaling in MFs inhibits the progression of hepatic fibrosis.

### Blocking of Notch signaling inhibits the activation of HSCs to MFs *in vitro*

**Figure 4:**
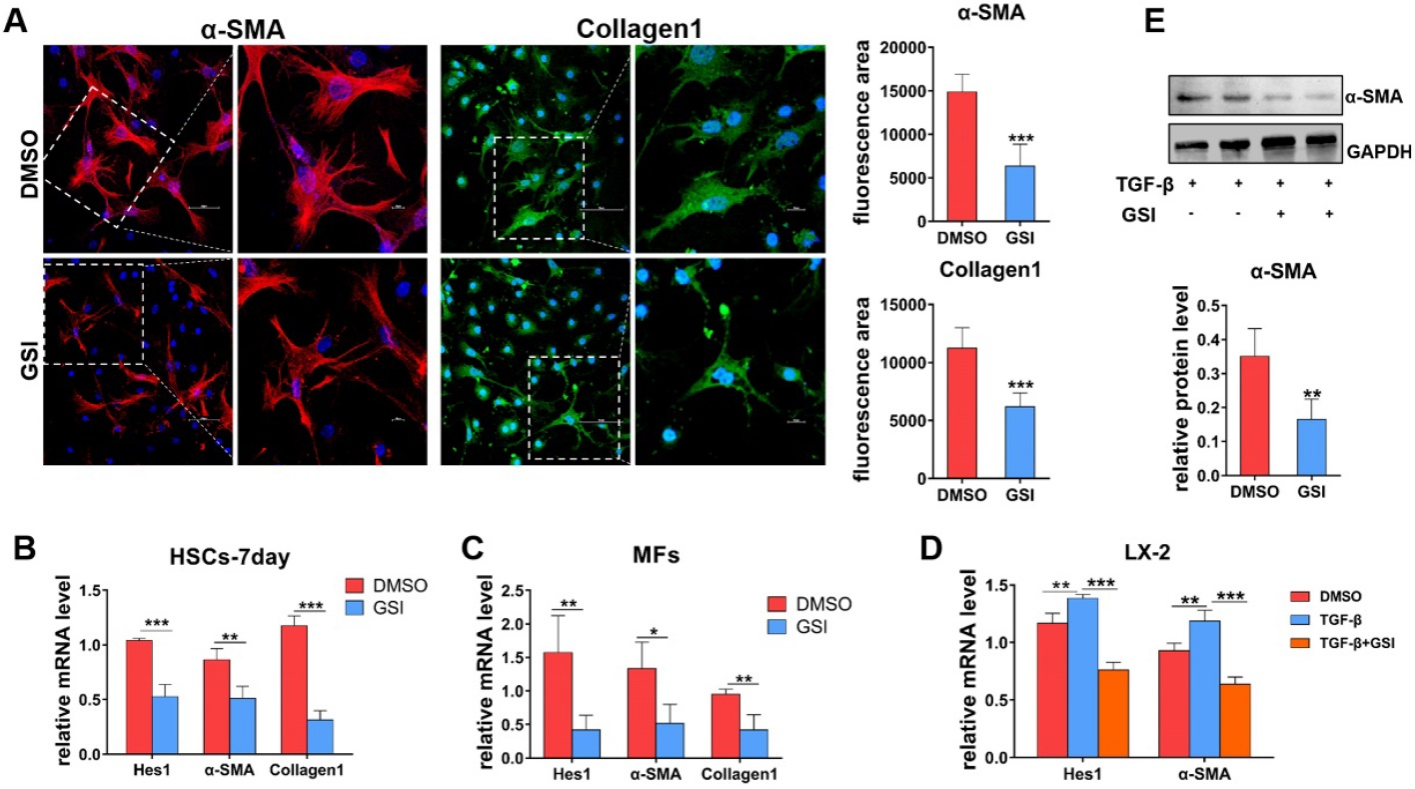
** In-vitro experiments verify the effect of Notch signaling blocking on HSCs/MFs.** (A) α-SMA and collagen1 IF staining of isolated primary liver MFs cultured *in vitro* and treated GSI for 24h from mice with hepatic fibrosis. (B) The mRNA level changes in isolated primary HSCs, cultured for 7 days and treated GSI for 24 hours, from normal mice. (C) The mRNA level changes of isolated MFs after GSI administration for 24 hours from mice with liver fibrosis. (D) The changes of mRNA level of human hepatic stellate cell line LX-2 treated GSI for 24 hours after activation administration via TGF-β. (E) Changes in LX-2 protein levels (n=6, *P < 0.05, **P < 0.01, ***P < 0.001).

To further delineate the role of Notch signaling in liver fibrosis progression, primary HSCs and hepatic MFs isolated from wild-type and CCl_4_-induced mice were cultured *in vitro*. Then, gamma(γ)-secretase inhibitor (GSI), a canonical Notch signaling repressor, was used to treat primary MFs and spontaneously activated HSCs (HSCs-7day, cultured for 7 days *in vitro*). As shown by IF staining results (Figure 4A), α-SMA and Collagen1 decreased in MFs after GSI treatment for 24h. qPCR also showed that the expression of α-SMA and Collagen1 was significantly reduced in HSCs-7day and MFs (Figure 4B, C). In addition, human hepatic stellate cell line LX-2 was stimulated with TGF-β for 48h for activation then treated with GSI, and the expression of activation marker was evaluated (Figure 4D). qPCR and Western bloting (WB) showed that α-SMA expression was modestly decreased in GSI pre-treated cells compared with cells without GSI pre-administration (Figure 4D, E). Collectively, these data further confirm that Notch disruption inhibits the activation of HSCs to MFs, as well as reduced ECM deposition.

### MFs-specific Notch signaling disruption promotes the regression process of liver fibrosis via expediting fibrous septum degradation and hepatocyte proliferation

**Figure 5:**
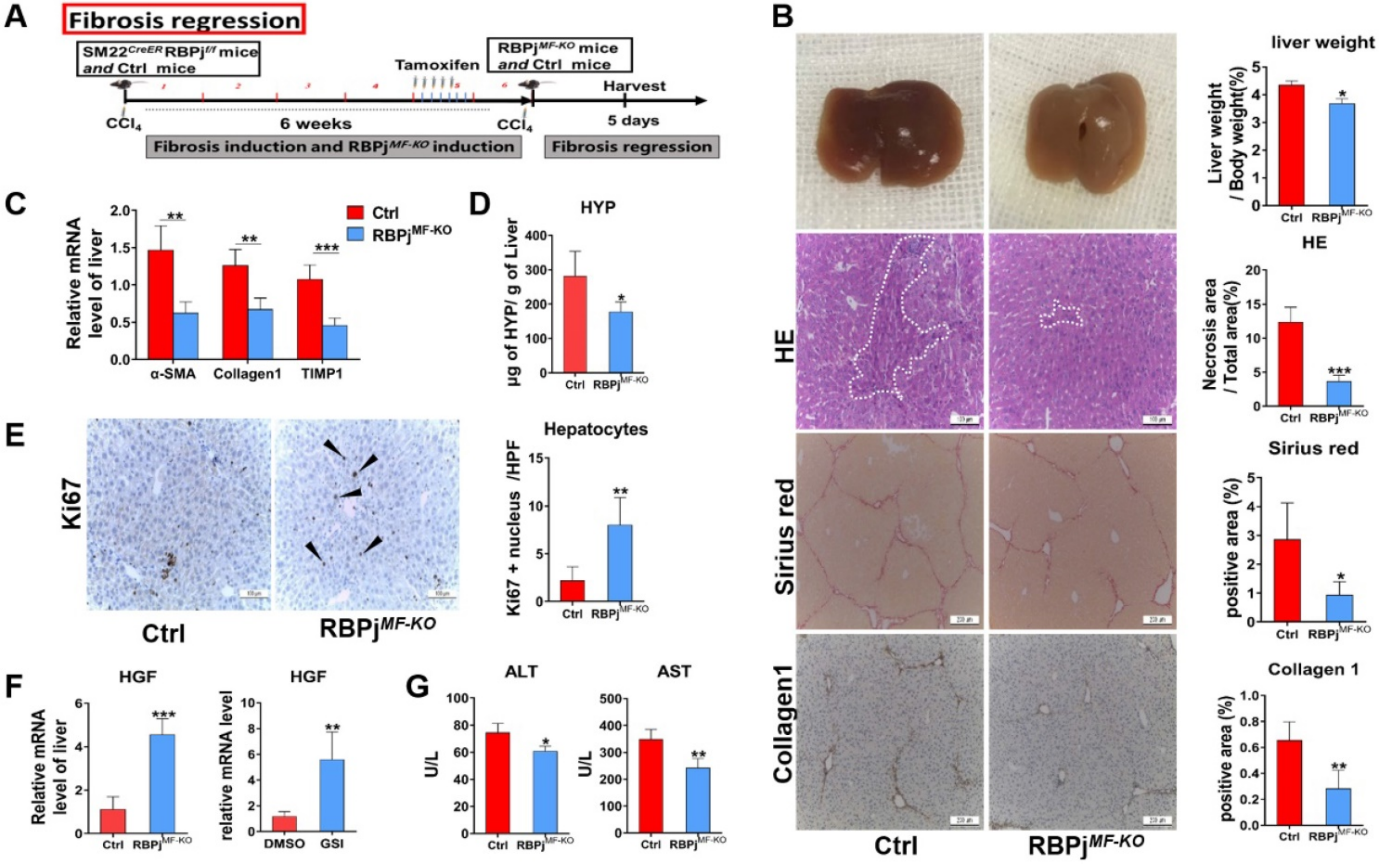
** Blocking of Notch signaling in MFs promotes the resolution of liver fibrosis.** (A) Induction schedule of liver fibrosis regression and RBPj knockout. (B) The general appearance of the liver, liver section HE, Sirius red and Collagen1 staining showed that ECM deposition was significantly reduced. (C) Changes in mRNA expression of fibrosis-related molecules in liver. (D) Changes in hydroxyproline level of liver tissues. (E) Liver section Ki67 staining showed that hepatocyte proliferation increased. (F) Detect the change of HGF mRNA level *in vivo* and the change of HGF mRNA level of MFs after treated GSI (100 μM for 24h) *in vitro*. (G) The serum liver function level was improved. (Bars = means ± SD, n=6, *P < 0.05, **P < 0.01, ***P < 0.001).

To further evaluate weather Notch signaling of MFs can mediate fibrosis regression models, we treated mice with CCl_4_ for 6 weeks to induce advanced liver fibrosis and allowed them to recover in the absence of CCl_4_ for 5 days 31 (Figure 5A). On gross observation, the livers of RBPj^MF-KO^ mice were better-perfused, smoother, and lighter in liver/body weight than those of control mice. HE staining demonstrated a significantly decreased tissue destruction area in RBPj^MF-KO^ mice comparing to control mice (Figure 5B), and RBPj^MF-KO^ mice also displayed reduced serum ALT and AST level (Figure 5G), together suggesting that blockade of Notch signaling benefits the recovery of liver tissue necrosis. In addition to the decrease of serum HYP level of RBPj^MF-KO^ mice (Figure 5D), fibrotic bridge and ECM deposition were markedly reduced in the liver section of RBPj^MF-KO^ mice compared with control mice, shown by Sirius Red and Collagen1 IHC staining (Figure 5B). Moreover, qPCR showed that α-SMA, Collagen1 and TIMP1 mRNA level decreased in liver of RBPj^MF-KO^ mice (Figure 5C), which indicates MFs-specific Notch inactivation expedited the degradation of fibrotic septa.

Next, we detected the condition of hepatocyte proliferation of control and RBPj^MF-KO^ mice by anti-Ki67 IHC staining. Anti-Ki67 IHC showed that Notch inactivation in MFs significantly enhanced hepatocyte proliferation in the process of liver fibrosis regression (Figure 5E). To clarify what triggers hepatocytes proliferation, the expression of several hepatocyte mitogens, including Wnt2a, Wnt9b, and HGF, was determined by qPCR. We observed the mRNA level of HGF was up-regulated in RBPj^MF-KO^ mice (Figure 5F). Moreover, we examined the mRNA level of HGF in apoptotic LX-2 (induced by being cultured with serum-free medium for 24h) in the attendance or absence of GSI administration. In line with this, HGF was remarkably increased in GSI-treated LX-2 as shown by qPCR (Figure 5F). These data indicated that Notch disruption in MFs may improve hepatocyte proliferation by enhancing the expression of HGF through other undefined signaling.

### Notch signaling disruption enhances apoptosis of MFs during the process of liver fibrosis regression

**Figure 6:**
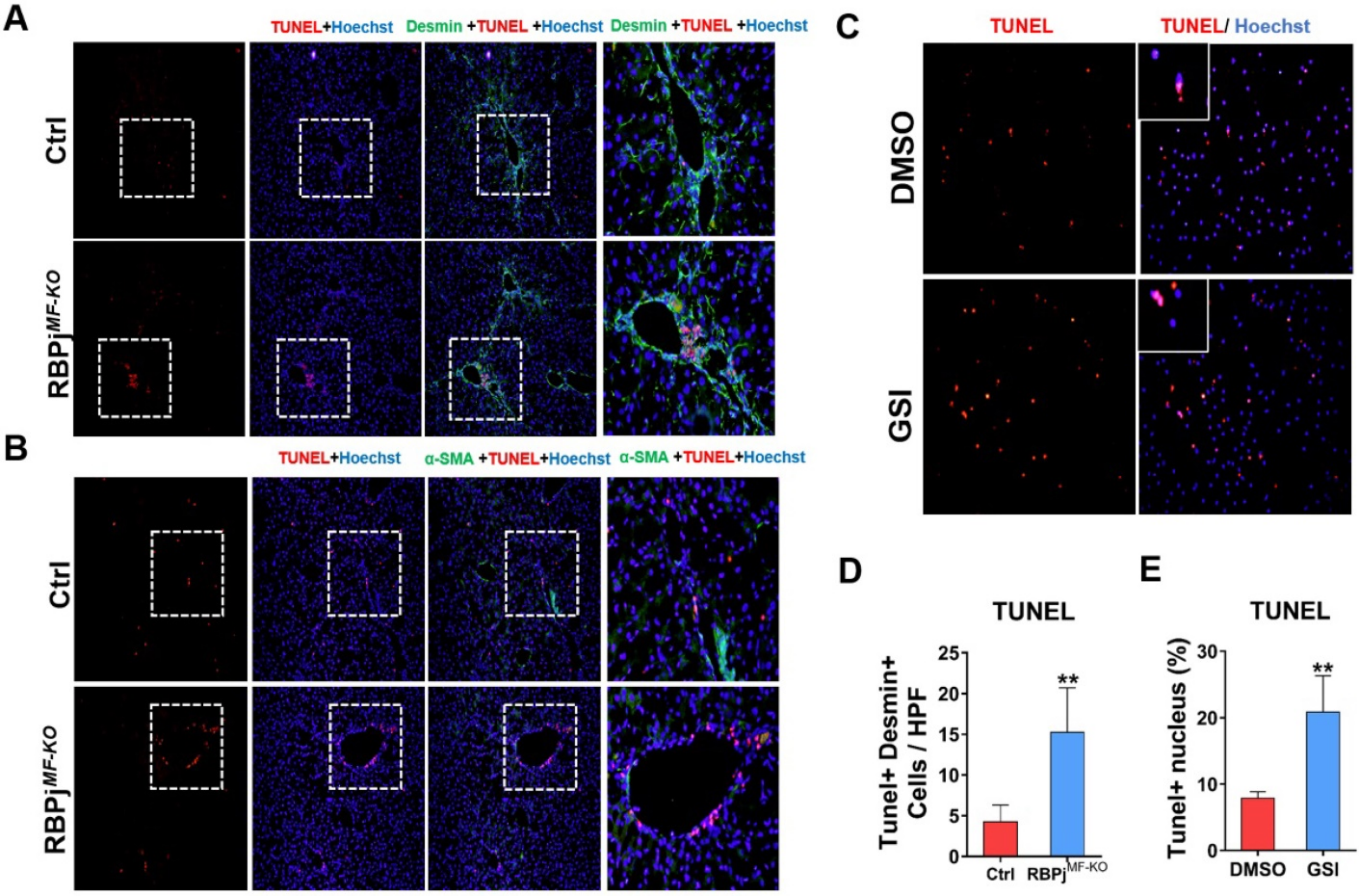
** Blocking of Notch signaling in MFs promotes MFs apoptosis during liver fibrosis regression.** (A) Liver TUNEL and Desmin co-localized staining, Desmin positive cells increased apoptosis. (B) Liver TUNEL and α-SMA co-localized staining. The expression of α-SMA reversed significantly, but the cell apoptosis in perivascular and portal area increased obviously. (C) TUNEL staining of culturing primary MFs after 24h treatment with GSI (100 μM for 24h) during the induction of apoptosis *in vitro*. (D) Statistical results of apoptosis in co-stained with TUNEL and Desmin. (E) Statistical results of apoptotic MFs after pro-apoptosis induction *in vitro*. (Bars = means ± SD, n=6, *P < 0.05, **P < 0.01, ***P < 0.001).

In the process of reversing liver fibrosis, MFs can undergo inactivation, aging or apoptosis to accomplish the fibrosis regression. Among them, the apoptosis of MFs makes a great contribution to the regression of liver fibrosis 6, 8. To explore whether Notch signaling is involved in MFs apoptosis during fibrosis regression, we performed co-localization staining of liver MFs markers α-SMA/Desmin and TUNEL in the liver section of control and RBPj^MF-KO^ mice (Figure 6A, B). The results showed that after Notch signaling was blocked, more positive apoptosis signals were observed in the α-SMA/Desmin positive area and perivascular area of liver (Figure 6D). After isolation from wild-type hepatic fibrosis mice, MFs were cultured in serum-free medium to induce apoptosis and treated with GSI for 24h, and we found TUNEL^+^ MFs significantly increased in GSI-treated cultures comparing to controls (Figure 6C, E). In summary, these data suggested that Notch signaling disruption favors regression of liver fibrosis through enhancing the apoptosis of MFs.

### Notch signaling blocking in MFs promotes Bax-mediated apoptosis of MFs by up-regulating Ngfr and Septin4

**Figure 7:**
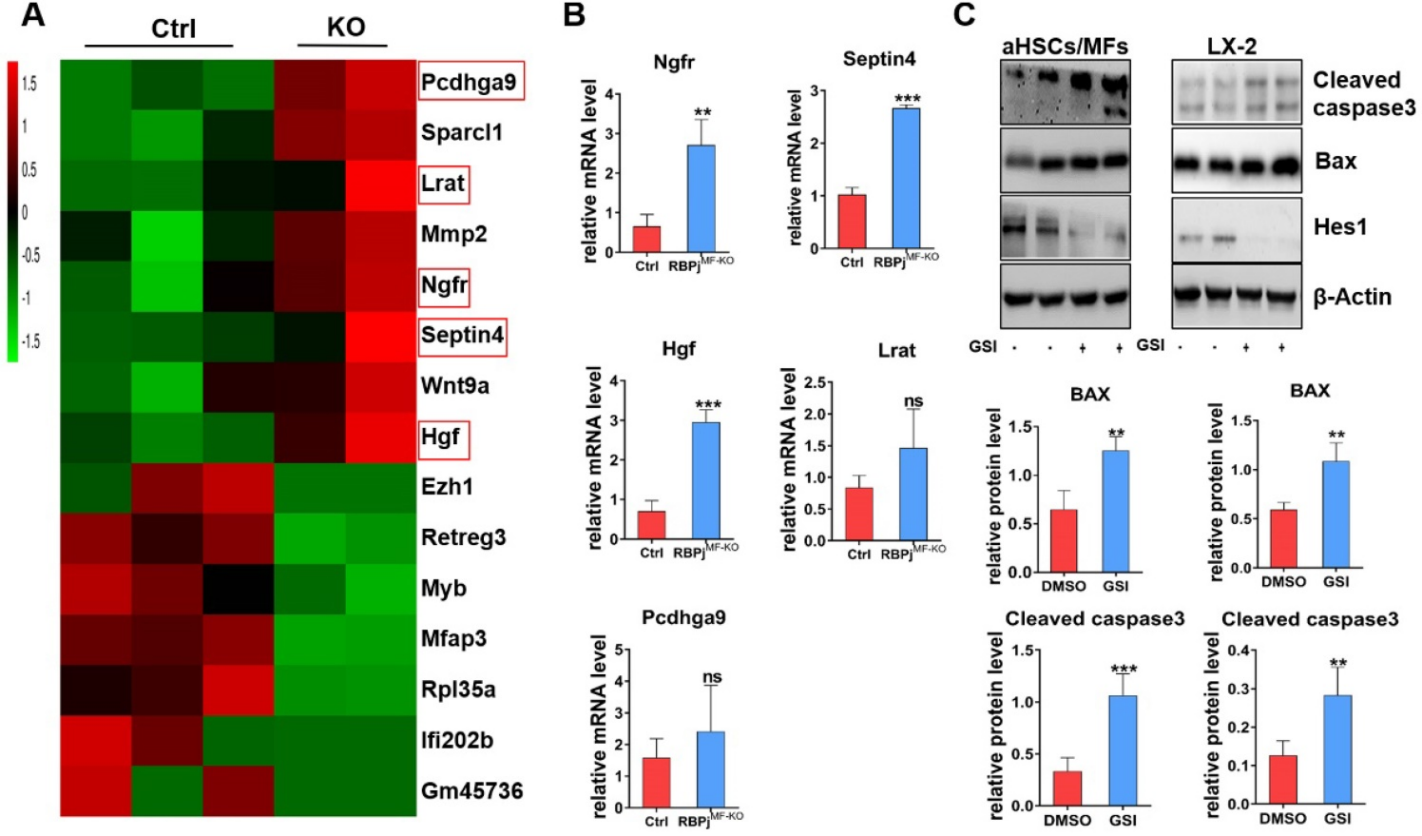
** In-vitro experiments verify that the blockade of Notch signaling in MFs promotes Bax-mediated apoptosis of MFs by potentially up-regulating Ngfr and Septin4.** (A) Heatmap comparison of related genes with RNA-sequencing data derived from RBPj^MF-KO^ and control MFs. (B) qPCR results of differentially expressed genes indicated that Ngfr, Septin4, HGF are consistent with the RNA-seq data, which may be involved in the mechanism of increased MFs apoptosis and increased hepatocyte proliferation. (n=3, *P < 0.05, ** *P* < 0.01, *** *P* < 0.001). (C) Primary isolated aHSCs/MFs and activated LX-2 cell line were cultured with serum-free medium for 24h to induce apoptosis and treated with GSI (100 μM for 24h) simultaneously. Protein levels of Hes1, BAX and Cleaved caspase3 were determined by WB and quantitatively compared. (Bars = means ± SD, n=6, *P < 0.05, **P < 0.01, ***P < 0.001).

To further investigate the mechanism of Notch signaling in regulating MFs apoptosis during the regression of liver fibrosis, we isolated the hepatic MFs in the reversal stage of liver fibrosis of RBPj^MF-KO^ and control mice and performed transcriptome sequencing. Comparison of gene expression profiles of MFs and further analysis of RNA-sequencing data showed that several apoptosis-related molecules, including nerve growth factor receptor (Ngfr) and Septin4, were up-regulated remarkably in RBPj^MF-KO^ mice (Figure 7A). qPCR confirmed up-regulation of Ngfr and Septin4 in MFs from RBPj^MF-KO^ mice (Figure 7B). Ngfr is known to mediate cell apoptosis, which is a member of tumor necrosis factor receptor (TNFR) superfamily 35-37. According to previous research 38, 39, overexpression of Ngfr in liver promotes the MFs apoptosis during regression of liver fibrosis. Septin4 belongs to Septin family and is involved in apoptosis, vesicle trafficking and other cellular events 40, 41. Using the *Septin4^-/-^* mice, Iwaisako et al. found that loss of Septin4 could aggravate the Collagens deposition 42. In our research, Ngfr and Septin4 were overexpressed after Notch inactivation in MFs (Figure 7A, B). Additionally, WB showed that Bax and Cleaved caspase3 were up-regulated after Notch signaling blockade in MFs and LX-2 during undergoing apoptosis (induced by being cultured with serum-free medium for 24h) (Figure 7C), which indicated that Notch downstream molecules may expedite the Ngfr and Septin4 expression in other mechanism to aggravate Bax-mediate apoptosis of MFs. Moreover, RNA-sequencing results showed that HGF was increased in RBPj^MF-KO^ (Figure 7A, B), which validated our previous data that Notch disruption in MFs enhances hepatocyte proliferation via up-regulating the expression of HGF during liver fibrosis regression (Figure 5E, F).

## Discussion

Notch signaling has been suspected for some time to play a role in liver fibrosis 43. Alagille syndrome patients (mutations in Jagged1 gene in 94% of patients or “Notch2” in 1-2%) were reported to rarely go onto cirrhosis and exhibit less ECM deposition than other chronic liver diseases, representing an early clue toward a role for Notch signaling pathway in the liver fibrotic response 30, 43, 44. Rat HSCs express Notch receptors *in vitro* and begin expressing Jagged1 upon activation and differentiation to MFs 25. Additionally, MFs activation and subsequent collagen deposition in the lung has also been found to be partially dependent on Notch activity, suggesting that the role for Notch in fibrosis is not limited to the liver 45. In the present study, MFs-specific Notch signaling is activated in liver fibrosis progression and depressed in liver fibrosis regression, which indicates that Notch signaling in MFs may participate in the regulation of liver fibrosis progression and regression (Figure 1). Then, we precisely confirmed the expression of one of the MFs markers- Sm22α, and utilized Sm22α^CreER^-RBPj^flox/flox^ mice after conditional induction to obtain a MFs-specific loss-of-function model of Notch signaling- RBPj^MF-KO^ (Figure 2). Indeed, Sm22α^CreER^ model has been included in MFs-related research 46, 47, however, the expression and pinpoint of Sm22α during the progression of liver fibrosis has not been determined. Based on our data (Figure 2A, B), Sm22α^CreER^ mice can be used as a reliable model for the study of hepatic MFs.

The GSI (pharmacological Notch signaling inhibitor) intraperitoneal injection alleviated liver fibrosis in rats 48. However, because of GSI's indiscriminate effect to all types of cells in the liver, it has not been elucidated which kind of cell Notch signaling plays an important role in this process. Our previous studies found that myeloid-specific disruption of RBPj ameliorated hepatic fibrosis and LSEC-specific Notch signaling activation aggravated liver fibrosis and inhibited hepatocyte proliferation 28, 29. Additionally, our recent research revealed that LSEC-specific Notch signaling disruption alleviated hepatic sinusoidal fibrosis (unpublished). However, the effects of MFs-specific Notch signaling on liver fibrosis are still ambiguous. Therefore, these prompted us to investigate the exact role and mechanism of MFs-specific Notch signaling to mediate liver fibrosis progression and regression. In the present study, specific blocking of Notch signaling of MFs inhibited the activation of HSCs to MFs both in cultures and RBPj^MF-KO^ mice, showing decreased expression of α-SMA and Desmin, the markers of HSCs-MFs activation (Figure 3, 4).

Another important effector mediating fibrosis is MMPs, which consist of a family of enzymes with different substrate affinities to matrix components 43, 49. These fibrinolytic mediators, especially MMP12 and MMP13, were mainly secreted by macrophages 50, other different MMPs were derived from MFs and neutrophils 49. In this study, the expression of MMP8, MMP9 and MMP12 increased in the liver of RBPj^MF-KO^ mice (Figure 3D). This is probably due to the increased MMPs expression of MFs. The blockage of Notch signaling in MFs not only reduced the deposition of ECM by inhibiting the activation of HSCs to MFs but also up-regulated the expression of MMPs to promote the degradation of ECM and thus to inhibit the progress of liver fibrosis. Besides, due to the Sm22α expression of hepatic vascular smooth muscle cells (VMSCs) in normal mice (Figure 2C), we also analyzed the phenotype of Sm22α^CreER^-RBPj^MF-KO^ and control mice without CCl_4_ administration. The results showed that the blockage of Notch signaling restricted to hepatic VMSCs had no effect on the liver of healthy mice (supplementary materials. Figure S1), which lays a theoretical foundation for the safety of targeting MFs-related Notch signaling to treat liver fibrosis in the future.

After the insults were terminated, liver fibrosis will be gradually reversed as mainly manifested of degradation of ECM and regeneration of liver tissues, which involves the interaction of many kinds of cells in the liver, such as LSECs, macrophages, MFs 6. The deactivation of MFs is key to fibrosis regression, and three mechanisms have been proposed: senescence, apoptosis and inactivation 6, 51. Indeed, in the present study, the regression of hepatic fibrosis was significantly improved in RBPj^MF-KO^ mice (Figure 5), which was mainly characterized by the decrease of extracellular matrix deposition (Figure 5B, D), the promotion of hepatocyte proliferation (Figure 5E) and the recovery of liver function (Figure 5G). These results were partly explained by the fact that Notch signaling regulated the expression of TIMP1 and HGF in MFs during fibrosis regression. Besides, liver section IF counterstaining and *in vitro* experiments showed that the MFs-specific Notch signaling disruption promoted the apoptosis of MFs (Figure 6), validating the conclusion of liver fibrosis regression acceleration in RBPj^MF-KO^ mice.

During fibrosis regression, senescence describes a phenotype of MFs with reduced fibrogenic gene expression and cell cycle exit, which confers susceptibility to natural killer (NK) cells mediated apoptosis 7. Driven by the withdrawal of anti-apoptotic signaling pathways as well as by NK, γδ T and CD8+ T cells, MFs undergo apoptosis 11, 51. Many signaling pathways have also been confirmed to stimulate apoptosis in activated HSCs-MFs. These include CEBP/β signaling, farnesoid X receptor (FXR or bile acid receptor), cannabinoid receptor 2 (CB2), adiponectin, Septin4 and TRAIL (also known as TNFSF10) 10, 40. Nerve growth factor (Ngf) can be released from infiltrating inflammatory cells or regenerating hepatocytes and is pro-apoptotic for activated HSCs-MFs 8, 38, 52. According to our RNA-sequencing results, blockade of Notch signaling in MFs upregulated the expression of Ngfr and Septin4. As a member of TNFR superfamily molecules, Ngfr mediated canonical cell apoptosis 53, 54. Previous study reported that in the fibrotic liver of mice with systemic Ngfr knockout, the apoptosis of MFs was significantly decreased and the regression of hepatic fibrosis was slowed down 39. Overexpression of Septin4 can ameliorate hepatic fibrosis in mice as well 55. So, in this study, the overexpression of Ngfr and Septin4 is likely the reason why Notch blockade in MFs promoted BAX-mediated apoptosis of MFs itself. However, the mechanism of Notch blockage leading to the increase of Ngfr and Septin4 remains to be explored.

In summary, the Notch signaling is closely associated with the liver fibrosis progression and regression. MFs-specific Notch signaling blockade can alleviate the liver fibrosis by suspending the progression of hepatic fibrosis, but also improving the resolution of hepatic fibrosis. As a major global healthcare burden, the discovery of key therapeutic targets with high relevance to liver fibrotic disease and the subsequent development of effective antifibrotic therapies directed against these targets continues to be a research priority. Our study establishes a relationship between MFs-specific Notch signaling and liver fibrosis progression and regression through Notch-mediated cytological and molecular changes, and sheds insights into therapeutic development against liver fibrosis targeting Notch signaling in MFs.

## Supplementary Material

Supplementary figure and table.

## Abbreviations

MFs: myofibroblasts
HSCs: hepatic stellate cells
α-SMA: α-smooth muscle actin
ECM: extracellular matrix
HGF: hepatocyte growth factor
MMPs: matrix metalloproteinases (MMPs)
TGF-β: transforming growth factor-β
PDGF-β: platelet-derived growth factor β
IL-17A: interleukin-17A
TNFR: tumor necrosis factor receptor
TIMPs: tissue inhibitors of metalloproteinases
NICD: Notch intracellular domain
RBPj: j-kappa recombination signal-binding protein
Sm22α: smooth muscle 22α
HYP: hydroxyproline
GSI: gamma(γ)-secretase inhibitor
Ngfr: nerve growth factor receptor
VSMCs: vascular smooth muscle cells
CCl4: carbon tetrachloride
IF: immunofluorescence
HE: hematoxylin and eosin
IHC: immunohistochemistry
SEM: scanning electron microscopy
