# Supplementary material for: Disruption of myofibroblastic Notch signaling attenuates liver fibrosis by modulating fibrosis progression and regression
Source: Int J Biol Sci. 2021 May 27;17(9):2135–46. doi: 10.7150/ijbs.60056 (PMC8241719; doi:10.7150/ijbs.60056)
Supplement: Supplementary file 1 — Supplementary figure and table. [file ijbsv17p2135s1.pdf]

Supplementary materials

Supplementary Table 1. Primers used for RT-PCR and genotyping.

Name	Forward (5'-3')	Reverse (5'-3')
Mouse Notch1	GATGGCCTCAATGGGTACAAG	TCGTTGTTGTTGATGTCACAGT
Mouse Notch2	GACTGCCAATACTCCACCTCT	CCATTTTCGCAGGGATGAGAT
Mouse Notch3	TGCCAGAGTTCAGTGGTGG	CACAGGCAAATCGGCCATC
Mouse Notch4	CTCTTGCCACTCAATTTCCCT	TTGCAGAGTTGGGTATCCCTG
Mouse Jagged1	CCTCGGGTCAGTTTGAGCTG	CCTTGAGGCACACTTTGAAGTA
Mouse Hes1	GATAGCTCCCGGCATTCCAAG	GCGCGGTATTTCCCCAACA
Mouse Hey1	CATGAAGAGAGCTCACCCAGA	CGCCGAACTCAAGTTTCC
Mouse RBPj	CACTGTTCAATCGCCTTC	AGTCACTGAGCACACAAG
Mouse β-actin	GGCTGTATTCCCCTCCATCG	CCAGTTGGTAACAATGCCATGT
Mouse TIMP1	ATGACTGGGGTGTAGGCGTA	TCAGAGCCAAAGCAGTGAGC
Mouse MMP8	TGCCACGATGGTTGCAGAG	AGGCATTTCCATAATCCCCATTG
Mouse MMP9	GCAGAGGCATACTTGTACCG	TGATGTTATGATGGTCCCACTTG
Mouse MMP12	GGGCTGCTCCCATGAATGAC	CCAGAGTTGAGTTGTCCAGTTG
Mouse MMP13	TGTTTGCAGAGCACTACTTGAA	CAGTCACCTCTAAGCCAAAGAAA
Mouse a-SMA	CCCAGACATCAGGGAGTAATGG	TCTATCGGATACTTCAGCGTCA
Mouse Collagen1	TAAGGGTCCCCAATGGTGAGA	GGGTCCCTCGACTCCTACAT
Mouse HGF	ATGTGGGGGACCAAACTTCTG	GGATGGCGACATGAAGCAG
Mouse Ngfr	CTAGGGGTGTCCTTTGGAGGT	CAGGGTTCACACACGGTCT
Mouse Septin4	TGAGCTGAGCAAGTTCGTGAA	ACAAGGAGCCTCTAAACTCCAC
Human Hes1	TCAACACGACACCGGATAAAC	GCCGCGAGCTATCTTTCTTCA
Human a-SMA	TTCATCGGGATGGAGTCTGCTGG	TCGGTCGGCAATGCCAGGGT
Human GAPDH	TTCATCGGGATGGAGTCTGCTGG	CTCCACGACGTACTCAGCG
Cre N1	CCGGTCGATGCAACGAGTGATGAGG	
Cre N2	GCCTCCAGCTTGCATGATCTCCGG	
R3	GTTCTTAACCTGTTGGTCGGAACC	
R4	GCTTGAGGCTTGATGTTCTGTATTGC	

PGKD	ACCGGTGGATGTGGAATGTGT

Supplementary Table 1. Antibodies used in this study.

Name	Supplier	Purpose
Anti-NICD	Abcam: ab8925	IHC
Anti-Collagen1	Abcam:ab34710	IHC
Anti- β -actin	Cell Signaling Technology	WB
Goat anti-Rabbit-HRP	Cell Signaling Technology	WB
Rabbit anti-Mouse-HRP	Cell Signaling Technology D3V2A	WB
Rabbit Anti-Hes1	Sigma：SAB2108472	IHC
Anti-Sm22α	Abcam：ab14106	IF
mouse Anti-a-SMA	Servicebio：GB13044	IF
mouse Anti-Desmin	Servicebio：GB12081	IF
Anti-F480	Ebioscience：14-4801-82	IF
Anti-Lyve	Relia Tech：103-M130	IF
Donkey anti-Rat Alexa fluo594	Invitrogen	IF
Goat anti-Rabbit FITC	Jackson Immuno Research	IF
Goat anti Rabbit Cy3	Jackson Immuno Research	IF
Goat anti Mouse Cy3	ServiceBio	IF
Anti-Hes1	Cell Signaling Technology	WB
Anti-Bax	Abcam：ab182733	WB
Anti-Cleaved Caspase3	Cell Signaling Technology	WB

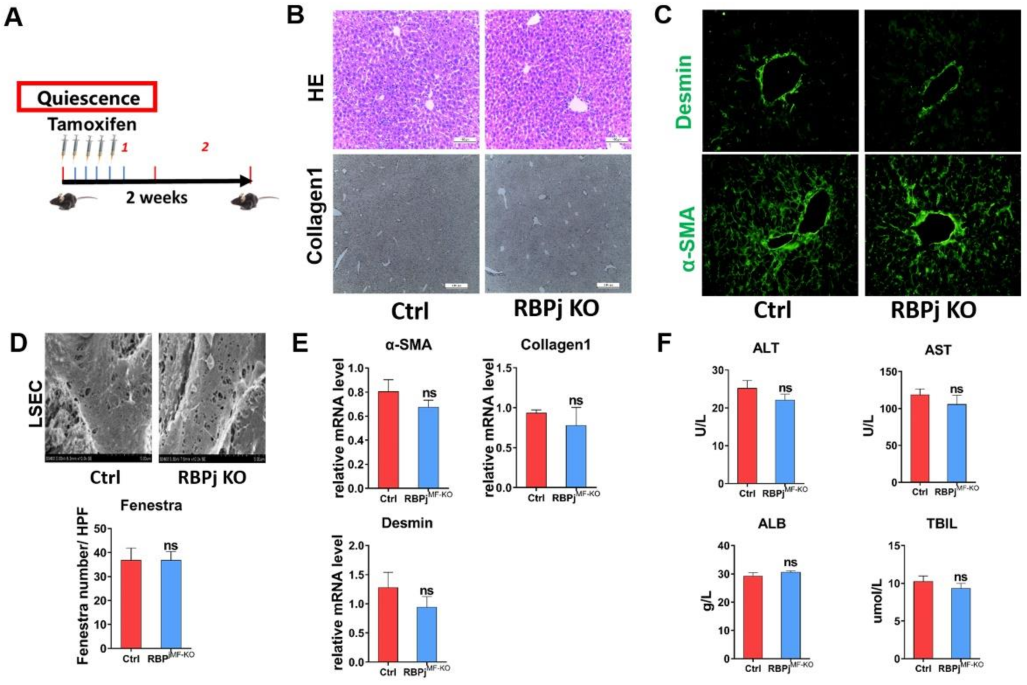

Supplementary Figure S1. Blockage of Notch signaling restricted to hepatic VMSCs has no effect on the liver of healthy mice. (A) Schedule of induction in **$Sm22\alpha^{CreER}$-$RBPj^{MF-KO}$** mice without CCl_4 administration. (B) HE and Collangen1 IHC staining of liver section. (C) IF staining of Desmin and α-SMA of liver section. (D) Fenestra of LSEC by SEM. (E) qPCR showed the relative mRNA level in liver. (F) Serum liver function by biochemical detection. (Bars = means ± SD, n=3, *$P < 0.05$, **$P < 0.01$, ***$P < 0.001$, ns, not significant).
